# FACS for Fungi: revealing population heterogeneity among fungal pathogens via flow cytometry

**DOI:** 10.1186/1476-9255-12-S1-O3

**Published:** 2015-04-16

**Authors:** Elizabeth R Ballou, Alistair JP Brown

**Affiliations:** 1 Aberdeen Fungal Group, School of Medical Sciences, University of Aberdeen, Institute of Medical Sciences, Foresterhill, AB25 2ZD, UK

## 

Although we often consider pathogens as presenting a single face to the immune system, it is clear that this “golf ball” model of pathogen-host interaction limits our understanding of population dynamics in the host. For example, others have shown that population heterogeneity in surface antigen presentation by *Trypanosoma brucei* and heterogeneity in the expression of pathogenicity factors by *Vibrio harveyi* and the human fungal pathogen *Candida albicans* are required for full virulence of these organisms [1-3]. This is perhaps counter-intuitive, given a working model in which virulence factor expression is correlated to degree of pathogenicity of a given organism. In this work, we discuss the application of flow cytometry techniques to the analysis of fungal pathogens. Specifically, we examine the impact of environmental fluctuations in nutrient availability on the presentation of cell wall components on the surface of fungal cells and use these features as markers for population heterogeneity. We demonstrate that population heterogeneity can be modulated by environmental conditions such as carbon source availability. We show that heterogeneity is rapidly lost when cells grown in a mixed nutrient environment representative of *in vivo* conditions are transitioned to the single nutrient conditions more typical of *in vitro* laboratory culture, and is slow to re-emerge when cells are transitioned back. Moreover, we show that heterogeneity in the presentation of pathogen-associated molecular patterns is similarly modulated by environmental conditions. Together, our observations underscore the potential for population-based analyses in investigating host-pathogen dynamics.
